# Genetic profile of sports climbing athletes from three different ethnicities

**DOI:** 10.5114/biolsport.2022.109958

**Published:** 2021-11-10

**Authors:** Mika Saito, Michał Ginszt, Ekaterina A. Semenova, Myosotis Massidda, Kinga Huminska-Lisowska, Monika Michałowska-Sawczyn, Hiroki Homma, Paweł Cięszczyk, Takanobu Okamoto, Andrey K. Larin, Edward V. Generozov, Piotr Majcher, Koichi Nakazato, Ildus I. Ahmetov, Naoki Kikuchi

**Affiliations:** 1 Graduate School of Health and Sport Science, Nippon Sport Science University, Tokyo, Japan; 2 Department of Rehabilitation and Physiotherapy, Medical University of Lublin, Poland; 3 Department of Molecular Biology and Genetics, Federal Research and Clinical Center of Physical-Chemical Medicine of Federal Medical Biological Agency, Moscow, Russia; 4 Research Institute of Physical Culture and Sport, Volga Region State University of Physical Culture, Sport and Tourism, Kazan, Russia; 5 Department of Life and Environmental Sciences, University of Cagliari, Italy; 6 Faculty of Medicine and Surgery, Graduate School of Exercise and Sports Sciences, University of Cagliari, Cagliari, Italy; 7 Faculty of Physical Education, Gdañsk University of Physical Education and Sport, Gdañsk, Poland; 8 Department of Physical Education, Plekhanov Russian University of Economics, Moscow, Russia; 9 Laboratory of Molecular Genetics, Kazan State Medical University, Kazan, Russia; 10 Research Institute for Sport and Exercise Sciences, Liverpool John Moores University, Liverpool, United Kingdom

**Keywords:** Sports Climbing, Polymorphism, ACTN3, ACE, CKM, TRHR

## Abstract

This study aimed to investigate the *ACTN3* R577X, *ACE* I/D, *CKM* rs8111989, and *TRHR* rs7832552 genotypes in climbers and controls in three ethnicities. The study consisted of 258 climbers (Japanese, n = 100; Polish, n = 128; Russian, n = 30) and 1151 controls (Japanese: n = 332, Polish: n = 635, Russian: n = 184). Genotyping results were analyzed using the TaqMan approach in Japanese and Polish subjects and HumanOmni1-Quad Bead Chips in Russian subjects. There were no significant differences in *ACTN3* R577X and *ACE* I/D polymorphism distribution between climbers and controls in any ethnic cohort or model. The frequencies of the C allele in the *CKM* polymorphism and the T allele in the *TRHR* polymorphism were higher in climbers than in controls only in the Russian cohort (p = 0.045 and p = 0.039, respectively). The results of the meta-analysis on three cohorts showed that the frequency of XX + RX genotypes in the *ACTN3* R577X polymorphism was significantly higher in climbers than that in the controls (p = 0.01). The X allele of the *ACTN3* R577X polymorphism was associated with sport climbing status, as assessed using a meta-analysis of climbers across three different ethnicities.

## INTRODUCTION

Sport climbing was selected for the Olympic Games Tokyo 2020 and will also be included in the Olympic Games Paris 2024. It includes three disciplines: speed climbing, bouldering, and lead climbing. The total time of climbing is approximately 6 min [1, 2], and competitive climbing is characterized by high-intensity intermittent exercise such as repeated climbing and rest periods in lead climbing and bouldering. Constant-speed climbing requires explosive power in a short time (less than 10 s). Bouldering and lead climbing have become popular and are performed indoors in climbing gyms and also outdoors. Previous studies have suggested that endurance, flexibility, grip strength relative to body mass, lean body mass percentage (% LBM), and fat mass percentage are related to climbing performance [3–9]. Elite climbers have significantly lower fat mass percentage [5, 8, 9] and take a longer time to reach exhaustion than those in non-elite climbers during endurance tests [4]; and have a higher grip strength relative to body mass and flexibility when compared to controls, non-elite climbers and elite climbers [3, 6]. Therefore, climbing performance is associated with various physical and morphological characteristics.

These physical and morphological characteristics are affected by environmental and genetic factors. Additionally, the heritability of athletic performance was recorded as being 66% in a twin study [10]. The R577X polymorphism in the α-actinin-3 gene (*ACTN3*) and the I/D polymorphism in the angiotensin-converting enzyme (*ACE*) are the most studied physical fitness-related genetic polymorphisms [11]. Moreover, muscle-specific creatine kinase and thyrotrophin-releasing hormone receptor (*TRHR*) polymorphisms have been reported to exhibit exercise-related phenotypes such as endurance performance and LBM [12–16]. These genetic variants may be associated with the climbing performance.

The *ACTN3* R577X polymorphism in the XX genotype is known as α-actinin-3 deficiency and is associated with lower composition of Type II fiber [17, 18], LBM [19] and strength [20], and higher flexibility [21, 22] and endurance capacity [23]. In addition, in both athletes and the general population. A meta-analysis reported that the frequency of the R allele was higher among power-oriented athletes than that in controls [11]. In addition, a previous study reported that a higher proportion of MHC-IIx% was observed in the XX genotype than that in the RR + RX genotype [17]. *ACE* I/D polymorphism was also reported in relation to fiber composition, and the proportion of MHC-I% was lower in the II genotype than that in the ID + DD genotype [17]. Furthermore, a meta-analysis reported that the II genotype was higher in endurance athletes than that in controls. *ACE* I/D polymorphism affects ACE activity, which is an important enzyme in the renin-angiotensin system, and lower ACE activity such as the I allele improves endurance performance [24]. Thus, these two gene polymorphisms have been investigated in association with power/sprint and endurance performance.

Previous studies have shown an association between *CKM* polymorphism (rs8111989) and CK activity after exercise and athlete status [12, 13]. Yuval et al. [25] reported that the frequency of the AA genotype was higher in individuals who exhibited an extreme increase in blood CK after exercise compared that in individuals with a normal response. Those with the AA genotype had a six-fold higher risk of having a high response of increase in blood CK compared to that in those with the GG and AG genotypes. Furthermore, a meta-analysis of *CKM* gene polymorphisms in case-control studies showed that the frequency of the GG genotype and G allele was higher in power-oriented athletes than that in controls [12]. In addition, the *TRHR* polymorphism was associated with fat mass percentage [26], and previous genome-wide association studies (GWAS) have shown that the *TRHR* polymorphism is related to lean body mass [15]. Cláudia et al. [16] suggested that the lean body mass was increasingly associated with the TT and CT genotypes than that with the CC genotype in older women.

The physical ability and morphological characteristics of climbers may be affected by these polymorphisms. However, evidence for an association between genetic polymorphisms and climbing performance is limited. We previously reported that the *ACTN3* R577X polymorphism is associated with climbing performance in a single ethnicity of Polish climbers [27]. Furthermore, we found an association between the *MCT1* T1470A polymorphism and lactate transport rate [28] and athletic status in Japanese and Polish climbers [29]; however, this association is not consistently confirmed among the two ethnicities. It is necessary to validate the evidence of genetic association in climbers by replication in independent and event-specific cohorts of climbers from different ethnicities. The aim of this study was to investigate *ACTN3* R577X, *ACE* I/D, *CKM* rs8111989, and *TRHR* rs7832552 genotypes in climbers and controls across three ethnicities, namely Japanese, Russian, and Polish.

## MATERIALS AND METHODS

### Subjects

The present study consisted of 258 climbers and 1151 controls. The study involving the Japanese athletes included 100 climbers (n = 66 males, n = 34 females; age 26.0 ± 9.5 years)—of which 60 were elite climbers—and 332 controls (n = 190 males, n = 142 females; age 56.5 ± 16.6 years). The study involving Polish athletes included 128 elite climbers (n = 98 males, n = 30 females; age 28.4 ± 6.0 years) and 635 controls (505 males, 130 females; age 25.1 ± 5.0 years). The study involving Russian athletes included 30 elite climbers (n = 13 females, n = 17 males; age 22.8 ± 0.8 years; all Caucasians) and 184 controls (n = 44 females, n = 140 males; age 44.9 ± 4.2 years; all Caucasians). All participants were informed of the purpose and methods of the study, and each one of them provided written informed consent for participation. The study was approved by the ethics committees of Nippon Sport Science University, Gdańsk University of Physical Education and Sport, and Federal Research and Clinical Center of Physical-Chemical Medicine of Federal Medical Biological Agency. The study was conducted in accordance with the Declaration of Helsinki for Human Research.

### Genotyping

#### Japanese climbers and controls and Polish climbers

Total DNA was extracted and isolated from the saliva of the participants (Japanese and Polish climbers and Japanese controls) using an Oragene-DNA Kit (DNA Genotek, Ontario, Canada). The *ACTN3* R577X (rs1815739), *ACE* I/D (rs4341), *CKM* (rs8111989), and *TRHR* (rs7832552) polymorphisms were genotyped using TaqMan SNP Genotyping Assay (Assay ID: *ACTN3* R577X: C____590093_1_, *ACE* I/D: C__29403047_10, *CKM* rs8111989: C___3145002_10, *TRHR* rs7832552: C__29085798_10) using a BioRad PCR System (CFD-3120J1, BioRad, Hercules, CA, USA). The genotyping mixture (total volume: 5 *μ*L) contained 2.5 *μ*L of GTXpress Master Mix, 0.125 *μ*L of assay mix (40×), and 1.375 *μ*L of distilled water with 1 *μ*L of genomic DNA (10 ng/*μ*L) per reaction. The thermal cycling conditions included an initial denaturation at 95°C for 20 s, followed by 40 cycles of denaturation at 95°C for 3 s and annealing/extension at 60°C for 20 s.

### Polish controls

DNA of Polish controls from *ACTN3* R577X, *ACE* I/D, CKM rs8111989, and *TRHR* rs7832552 polymorphisms were extracted from the buccal cells using a High Pure PCR Template Preparation Kit (Roche, Switzerland) according to the manufacturer’s instructions. The genotyping mixture (total volume: 5 *μ*L) contained 2.5 *μ*L of TaqPath ProAmp Master Mix (ThermoFisher Scientific, Germany), 0.25 *μ*L of assay mix (10X), and 1 *μ*L of distilled water with 1.25 *μ*L of genomic DNA (˜10 ng/*μ*L) per reaction. The thermal cycling conditions included a pre-read at 60°C for 30 s, an initial denaturation at 95°C for 5 min, followed by 40 cycles of denaturation at 95°C for 5 s and annealing/extension at 60°C for 30 s, and a post-read at 60°C for 30 s. Genotyping reactions were performed on a CFX Connect Real-Time PCR Detection System (BioRad, USA).

### Russian climbers and controls

In Russian climbers and controls, molecular genetic analysis was performed using DNA samples obtained from leukocytes (venous blood). Venous blood samples (4 mL) were collected in tubes containing ethylenediaminetetraacetic acid (EDTA) (Vacuette EDTA tubes, Greiner Bio-One, Austria). Blood samples were transported to the laboratory at 4°C, and DNA was extracted on the same day. DNA extraction and purification were performed using a commercial kit according to the manufacturer’s instructions (Technoclon, Russia) and included chemical lysis, selective DNA binding on silica spin columns, and ethanol washing. The quality of the extracted DNA was assessed using agarose gel electrophoresis. HumanOmni1-Quad BeadChips (Illumina Inc., USA) were used for genotyping 1,140,419 SNPs (including *ACTN3*, *ACE*, *CKM*, and *TRHR* polymorphisms) in athletes and controls. The assay required 200 ng of DNA sample as input at a concentration of at least 50 ng/*μ*L. The exact concentrations of DNA in each sample were measured using a Qubit Fluorometer (Invitrogen, USA). All further procedures were performed according to the instructions of the Infinium^®^ HD Assay (Illumina, USA).

### Statistical analyses

Genotype and allele frequencies were calculated for all gene polymorphisms, and the Hardy–Weinberg equilibrium was assessed using the *X*2 test. In addition, a meta-analysis of three ethnic cohorts was conducted to investigate the association between each gene polymorphism frequency of climbers and controls. Meta-analysis was conducted using the Review Manager software program (version 5.3; Copenhagen: The Nordic Cochrane Center, The Cochrane Collaboration, http://tech.cochrane.org/revman). The level of significance was set at P < 0.05.

## RESULTS

The *ACTN3* R577X, *ACE* I/D, *CKM* rs8111989, and *TRHR* rs7832552 polymorphisms among climbers and controls were in Hardy–Weinberg equilibrium. There were no significant differences in the distribution of *ACTN3* R577X and *ACE* I/D polymorphisms between climbers and controls in any ethnic cohort or model (Table 1). The frequencies of the C allele in the *CKM* polymorphism and the T allele in the *TRHR* polymorphism were higher in climbers than those in controls only in the Russian cohort (Table 1, *CKM*; T allele vs. C allele, p = 0.045, *TRHR*; C allele vs. T allele, p = 0.039).

**TABLE 1:** Frequency of ACTN3 R577X, ACE I/D, CKM rs8111989 and TRHR rs7832552 polymorphism in Japanese, Polish, and Russian climbers and controls.

		Climbers					Controls					P value	
				Genotype n (%)		Allele n (%)		Genotype n (%)			Allele n (%)	Genotype	Allele
ACTN3		n	RR	RX	XX	X	n	RR	RX	XX	X		
	Japanese	99	14(14)	56(57)	29(29)	114(58)	332	73(22)	169(51)	90(27)	349(53)	0.232	0.214
	Polish	125	41(33)	63(50)	21(17)	105(42)	635	256(40)	269(43)	110(17)	489(39)	0.215	0.300
	Russian	30	8(27)	17(56)	5(17)	27(45)	184	67(36)	72(39)	45(25)	162(44)	0.194	0.887

ACE			II	ID	DD	D		II	ID	DD	D		
	Japanese	97	44(46)	43(44)	10(10)	63(32)	332	129(39)	156(47)	47(14)	250(38)	0.419	0.188
	Polish	121	29(24)	64(53)	28(23)	120(50)	634	148(23)	312(49)	174(28)	660(52)	0.607	0.482
	Russian	30	9(30)	14(47)	7(23)	28(47)	184	55(30)	91(49)	38(21)	167(45)	0.937	0.853

CKM			TT	CT	CC	C		TT	CT	CC	C		
	Japanese	89	72(81)	16(18)	1(1)	18(10)	332	251(75)	69(21)	12(4)	93(14)	0.379	0.173
	Polish	122	45(37)	65(53)	12(10)	89(36)	635	283(45)	279(44)	73(11)	425(33)	0.164	0.363
	Russian	27	10(37)	12(44)	5(19)	22(41)	184	97(53)	73(40)	14(7)	101(27)	0.112	0.045
TRHR			CC	CT	TT	T		CC	CT	TT	T		
	Japanese	94	30(32)	33(35)	31(33)	95(51)	332	115(35)	138(41)	79(24)	296(45)	0.191	0.148
	Polish	126	66(52)	50(40)	10(8)	70(28)	635	309(49)	264(41)	62(10)	388(31)	0.685	0.381
	Russian	30	11(37)	15(50)	4(13)	23(38)	184	103(56)	68(37)	13(7)	94(26)	0.122	0.039

**FIG. 1:**
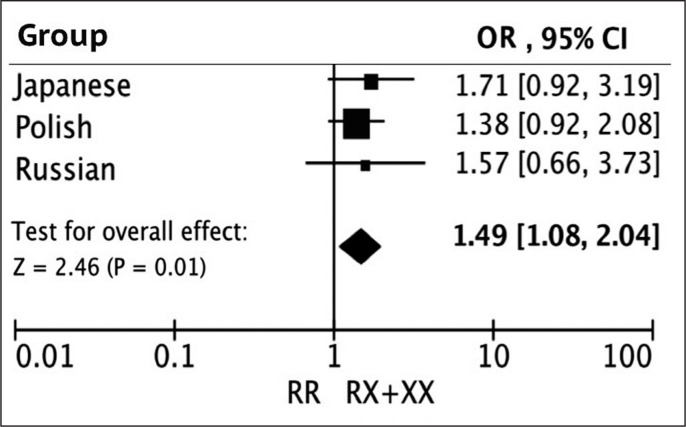
The results of meta-analysis of three ethnic cohorts for the association between *ACTN3* R577X (RR vs. RX + XX) polymorphism and climbing status.

The results of the meta-analysis across the three cohorts showed that the frequency of the XX + RX genotypes in the *ACTN3* R577X polymorphism was significantly higher in Japanese, Polish, and Russian climbers than that in the controls (Table 2, Fig 1, odds ratio: 1.49, 95%CI: 1.08–2.04, p = 0.01).

**TABLE 2:** The results of meta-analysis of three ethnic cohorts for the association between *ACTN3* R577X, *ACE* I/D, *CKM* rs8111989, and *TRHR* rs7832552 polymorphism and climbing status.

	Minor allele	Dominant			Recessive			Allele		
		OR	95%CI	P value	OR	95%CI	P value	OR	95%CI	P value
ACTN3	X	0.98	0.70–1.37	0.91	1.49	1.08–2.04	0.01	1.17	0.96–1.42	0.12
ACE	D	0.82	0.57–1.17	0.27	0.88	0.65–1.18	0.39	0.88	0.72–1.08	0.23
CKM	C	1.03	0.60–1.77	0.9	1.21	0.89–1.64	0.23	1.12	0.89–1.41	0.35
TRHR	T	1.31	0.90–1.93	0.16	1.06	0.80–1.41	0.69	1.12	0.91–1.37	0.29

According to the meta-analysis, the dominant, recessive and allele frequency distribution of the *ACE* I/D (dominant: odds ratio: 0.82, 95%CI: 0.57–1.17, p = 0.27, recessive: odds ratio: 0.88, 95%CI: 0.65–1.18, p = 0.39, allele: odds ratio: 0.88, 95%CI: 0.72–1.08, p = 0.23, respectively), *CKM* rs8111989 (dominant: odds ratio: 1.03, 95%CI: 0.60–1.77, p = 0.90, recessive: odds ratio: 1.21, 95%CI: 0.89–1.64, p = 0.23, allele: odds ratio: 1.12, 95%CI: 0.89–1.41, p = 0.35 respectively), and *TRHR* rs7832552 (dominant: odds ratio 1.31:, 95%CI: 0.90–1.93, p = 0.16, recessive: odds ratio: 1.06, 95%CI: 0.80–1.41, p = 0.69, allele: odds ratio: 1.12, 95%CI: 0.91–1.37, p = 0.29, respectively) polymorphisms among climbers were not significantly different compared those of the controls.

## DISCUSSION

This is the first study to investigate the genetic profiles of sports climbing athletes across different ethnicities. Our results showed that the *ACTN3* X allele (both RX and XX genotypes) was associated with climbing status (odds ratio: 1.49, 95%CI: 1.08–2.04, p = 0.01). Previously, the *ACTN3* RR genotype was reported to be significantly higher frequency in 50 boulderers than that in combined 50 lead climbers and 100 controls, but no significant difference in genotype frequency in *ACTN3* R577X between boulderers and controls was reported [27]. The current study included a total of 258 climbers and 1151 controls, which might be sufficient to confirm the relationship between the genetic polymorphisms and climbing performance compared with the sample sizes of previous studies [27]. Conversely, the meta-analysis results showed no significant differences in the genotype and allele distribution of the *ACE* I/D, *CKM* (rs8111989), and *TRHR* (rs7832552) polymorphisms between climbers and controls (Table 2). However, a higher frequency of the C allele in the *CKM* polymorphism and T allele in the *TRHR* polymorphism was observed in Russian climbers compared with that in the controls (p = 0.045 and p = 0.039, respectively).

Our results from the meta-analysis showed that the X allele (RX and XX genotypes) of the *ACTN3* R577X polymorphism, which reported a negative association of power-oriented athlete status and/or performance [11], was associated with the climbing status. We have reported in a previous study that individuals with the RX + XX genotype in the *ACTN3* R577X polymorphism had higher flexibility than those with the RR genotype [21] [30], and this could be one of the positive mechanisms determining climbing performance. These results supported that gymnasts that require higher flexibility and have *ACTN3* RR and RX genotypes show lower athletic performance than those with the XX genotype [31]. However, others have reported no association with female rhythmic gymnastics [32]. Broos et al. [33] reported that individuals with the RR genotype of the ACTN3 R577X polymorphism have higher elasticity of single fibers as measured by hysteresis and Young’s modulus compared to those in individuals with RX and XX genotypes among subjects with spinal cord injury. Seto et al. [34] reported that titin, which is related to muscle elasticity, showed preferential interaction with α-actinin-2 instead of α-actinin-3. These studies indicated that the absence of α-actinin-3 may be associated with muscle elasticity in humans. Therefore, the RX and XX genotypes in *ACTN3* may be associated with climbing performance due to the higher potential of flexibility, which is one of the factors determining elite performance in climbers, according to previous studies [3, 6, 7].

Second, in *ACTN3* KO mice, oxidative metabolism and recovery from contraction-induced fatigue were greater than those in WT mice [35]. These results suggest that *ACTN3* deficiency enhances the endurance capacity. A previous study on endurance performance in climbers reported that bent arm hang time, maximum pull-ups, and VO_2max_ are strongly correlated with climbing ability [7]. Furthermore, Espana-Romero et al. suggested that elite climbers had a significantly longer time to exhaustion than that did non-elite climbers during endurance tests determining volitional fatigue on a vertical climbing ergometer [4]. Endurance capacity, such as contentious force production and recovery after climbing, may be important for both lead climbers and boulderers, because climbing competition has high-intensity intermittent characteristics. However, the association between the *ACTN3* R577X polymorphism and endurance performance is inconsistent in humans [11]. Future studies are needed to validate the association between *ACTN3* R577X and endurance capacity in athletes.

A recent meta-analysis suggested that the I allele of the *ACE* I/D polymorphism is advantageous for endurance athletes [11]. Endurance athletes’ carrying the I allele showed higher VO_2max_ than that in athletes who were D allele carriers [36]. Concerning the mechanism of the effect of *ACE* I/D polymorphism on endurance capacity, a previous study suggested that capillary-to-fiber ratio and capillary density in the vastus lateralis were higher in athletes with the I allele than in athletes with the DD genotype, and the pro-angiogenic protein VEGF level in the vastus lateralis was the same [36]. In addition, Vaghan et al. [37] reported that carriers of the I allele showed a higher fold change in subsarcolemmal mitochondria in the vastus lateralis after 6 weeks of supervised bicycle exercise compared with non-carriers of the I allele. However, our results showed a lack of association between the *ACE* I/D genotype and climbing status.

In the present study, the frequency of the G allele of the *CKM* polymorphism and the T allele of the *TRHR* polymorphism was higher in climbers than those in controls only in the Russian cohort. Previous studies have shown that the frequency of the G allele and GG genotype of the *CKM* polymorphism was higher in sprint/power athletes than that in controls [12]. *CKM* polymorphism (rs8111989) is located in the 3’ untranslated region and may influence mRNA stability and change gene expression. Interestingly, *CKM* polymorphism (rs8111989) regulates *MARK4* gene expression, which may be associated with protein synthesis in skeletal muscle [38].

Subjects with the C allele of the *TRHR* polymorphism showed over 2-fold higher risk than that did those with TT genotype for sarcopenia [39]. A genome-wide association study indicated that lean body mass in T-allele carriers was higher than that in C allele carriers. Furthermore, the T-allele has been associated with higher sprint/power performance [30]. In the present study, the association between *CKM* and *TRHR* polymorphisms was observed only in the Russian cohort. The G allele of the *CKM* polymorphism and T allele of the TRHR polymorphism have been related to power-oriented characteristics [12, 30]. Regarding the sample analyzed here, Russian climbers included a higher percentage of speed climbers than that did the Japanese and Polish cohorts (speed climbers: Russian, 40%; Japanese, 1%; Polish, 5%). Speed climbers require explosive power and strength, which is different from the qualities required in lead climbing and bouldering athletes. The effects of these four polymorphisms (*ACTN3*, *ACE*, *CKM*, and *TRHR*) and environmental factors may interact and influence climbing performance.

The limitations of present study are that *ACTN3* R577X polymorphism shows no significant difference in each three ethnicity. In addition, *CKM* rs8111989 and TRHR rs7832552 polymorphism could not replicate in Japanese and Polish climbers. Further research needs to consider relationship with these gene polymorphisms with more sample size and several ethnic. Furthermore, To uncover these multiple genetic effects, the application of high-throughput technologies, such as genome-wide association studies or next-generation whole genome and/or exome sequencing, and total genetic score (TGS) of performance-related variants [40] including MCT1 T1470A polymorphism which we reported association with climbers [29] will be necessary.

## CONCLUSIONS

In summary, the X allele of the *ACTN3* R577X polymorphism was associated with sport climbing status, as assessed by a meta-analysis of climbers from three different ethnicities. However, further replication and functional studies are necessary to confirm these findings.
